# The Effect of Pleural Abrasion on the Treatment of Primary Spontaneous Pneumothorax: A Systematic Review of Randomized Controlled Trials

**DOI:** 10.1371/journal.pone.0127857

**Published:** 2015-06-04

**Authors:** Zhou-Gui Ling, Yan-bin Wu, Mo-yu Ming, Shuang-qi Cai, Yi-Qiang Chen

**Affiliations:** 1 Institute of Respiratory Diseases, the First Affiliated Hospital of Guangxi Medical University, Nanning, Guangxi, China; 2 Department of Respiratory Diseases, the Fourth Affiliated Hospital of Guangxi Medical University, Liuzhou, Guangxi, China; Baylor College of Medicine, UNITED STATES

## Abstract

**Background:**

Pleural abrasion has been widely used to control the recurrence of primary spontaneous pneumothorax (PSP). However, controversy still exists regarding the advantages and disadvantages of pleural abrasion compared with other interventions in preventing the recurrence of PSP.

**Methods:**

The PubMed, Embase, and Cochrane Central Register of Controlled Trials databases were searched up to December 15, 2014 to identify randomized controlled trials (RCTs) that compared the effects of pleural abrasion with those of other interventions in the treatment of PSP. The study outcomes included the PSP recurrence rate and the occurrence rate of adverse effects.

**Results:**

Mechanical pleural abrasion and apical pleurectomy after thoracoscopic stapled bullectomy exhibited similarly persistent postoperative air leak occurrence rates (p = 0.978) and 1-year PSP recurrence rates (p = 0.821), whereas pleural abrasion led to reduced residual chest pain and discomfort (p = 0.001) and a smaller rate of hemothorax (p = 0.036) than did apical pleurectomy. However, the addition of minocycline pleurodesis to pleural abrasion did not reduce the pneumothorax recurrence rate compared with apical pleurectomy (3.8% for both procedures) but was associated with fewer complications. There was no statistical difference in the pneumothorax recurrence rate between mechanical pleural abrasion and chemical pleurodesis with minocycline on either an intention-to-treat basis (4 of 42 versus 0 of 42, p = 0.12; Fisher exact test) or after exclusions (2 of 40 versus 0 of 42, p = 0.24; Fisher exact test). Pleural abrasion plus minocycline pleurodesis also did not reduce the pneumothorax recurrence rate compared with pleural abrasion alone (p = 0.055). Moreover, pleural abrasion plus minocycline pleurodesis was associated with more intense acute chest pain. The postoperative overall recurrence rate in patients who underwent staple line coverage with absorbable cellulose mesh and fibrin glue was similar to that with mechanical abrasion after thoracoscopic bullectomy (13.8% vs. 14.2%, respectively; p = 0.555), but staple line coverage resulted in less postoperative residual pain than mechanical abrasion (0.4% vs.3.2%; p<0.0001). Pleural abrasion after thoracoscopic wedge resection did not decrease the recurrence of pneumothorax compared with wedge resection alone (p = 0.791), but the intraoperative bleeding and postoperative pleural drainage rates were higher when pleural abrasion was performed.

**Conclusions:**

In addition to resulting in the same pneumothorax recurrence rate, thoracoscopic pleural abrasion with or without minocycline pleurodesis is safer than apical pleurectomy in the treatment of PSP. However, minocycline pleurodesis with or without pleural abrasion is not any more effective than pleural abrasion alone. Moreover, additional mechanical abrasion is not safer than additional staple line coverage with absorbable cellulose mesh and fibrin glue after thoracoscopic bullectomy because of increased postoperative pain. Additionally, pleural abrasion after thoracoscopic wedge resection should not be recommended for routine application due to the greater incidence of adverse effects than wedge resection alone. However, further large-scale, well-designed RCTs are needed to confirm the best procedure.

## Introduction

Primary spontaneous pneumothorax (PSP) most commonly occurs in young patients with no apparent underlying lung disease [1]. The main characteristic of PSP is its great tendency to recur. Simple aspiration and chest-tube drainage are often used to treat PSP, with a recurrence rate at 1 year of approximately 30% (range 16–52) [2–4]. Thus, surgical management is required to decrease the recurrence of PSP. Since the 1990s, video-assisted thoracoscopic surgery (VATS) has become the preferred treatment to decrease the recurrence of PSP; however, even with this surgery, PSP recurs in 5% to 10% of cases [5]. Persistent and recurrent pneumothorax is a clinical entity that requires pleurodesis to avoid the accumulation of air in the pleural space [6]. To successfully perform pleurodesis, complete apposition between the visceral and parietal pleura is needed [7]. The operative methods used to prevent recurrence include partial pleurectomy, chemical or mechanical pleurodesis and wide staple line coverage with absorbable material [8]. Mechanical pleural abrasion induces adhesion between the visceral and parietal pleura due to mechanical irritation [9, 10].

Although pleural abrasion is one of the most widely used techniques of pleurodesis, there is little evidence that this method actually reduces the recurrence of PSP [11]. The British Thoracic Society guidelines indicate that the recurrence rate with pleural abrasion is higher than that with pleurectomy [12]. Recently, a retrospective study indicated that the risk of recurrence after surgery in PSP was significantly lower in patients who underwent a thoracoscopic wedge resection with parietal pleurectomy than in patients who underwent pleural abrasion [13]. Another trial also showed that the use of talc pleurodesis with pleural abrasion may not decrease the incidence of prolonged air leak in the VATS spontaneous pneumothorax operation [14]. In a nonrandomized trial, however, Lee et al. noted that coverage with a polyglycolic acid sheet and pleural abrasion after thoracoscopic bullectomy is effective for preventing prolonged postoperative air leaks and reducing postoperative recurrence rates [15]. In addition, one RCT found that pleural abrasion in the treatment of PSP had low morbidity and a low rate of hemothorax postoperatively compared with apical pleurectomy, and no difference in the later recurrence rate was observed between the two procedures [16]. Thus, controversy remains regarding the effects of pleural abrasion in the treatment of PSP. Consequently, we performed a systematic review of published randomized controlled trials (RCTs) to evaluate the advantages and disadvantages of pleural abrasion compared with other interventions in preventing the recurrence of PSP.

## Methods

### Search strategy and study selection

Portions of the descriptions of the methods were taken from two articles [17, 18]. Two authors (ZGL and YBW) independently searched the PubMed, Embase, and Cochrane Central Register of Controlled Trials (CENTRAL) databases for relevant RCTs published through December 15, 2014. The search keywords included “pleural abrasion,” “mechanical pleurodesis” and “pneumothorax,” and a search was conducted for articles containing these keywords in the title, abstract, with no limits on the publication dates. Articles were also identified using the related-articles function in PubMed. The identified articles’ references were searched manually, as well. This process was performed repeatedly until no additional articles could be found. Although no language restrictions were applied initially, the full-text review and final analysis were limited to English-language articles. Additionally, trials with small sample sizes (n<10) were excluded to avoid selection bias.

### Eligibility criteria

We included full-text publications that examined pleural abrasion alone or in combination with other interventions for the treatment of PSP patients. We limited our selected RCTs to those that investigated only human subjects. Non-randomized trials, case-control studies, cohort studies and retrospective studies were excluded because we were concerned that they would not provide sufficiently high-quality evidence. When there was evidence that some study subjects may have been investigated in more than one study, the studies in question were discussed by ZGL, YBW, and YQC, and only the best-quality study among these studies was selected for inclusion in our study. Two reviewers (SQC and YQC) independently judged study eligibility while screening the citations. Disagreements were resolved by discussion and consensus.

### Data extraction and quality assessment

Two investigators (ZGL and YBW) independently extracted or computed the following data for each study: the first author, the year of publication, the study location, the number of patients, the study design, the quality score, intervention details and outcomes (including the PSP recurrence rate and other surgical outcomes). Another author (SQC) checked these data. Any disagreements were resolved by discussion and consensus. When any of the information needed was missing from a study, an attempt was made to contact the authors of that study to obtain the missing data.

Two non-blinded investigators (MYM and YQC) performed a quality assessment of the included studies using a modified 10-point scoring system as described previously [19]. Differences in scoring were resolved by discussion and consensus.

## Results

### Study identification and selection

**Fig 1:**
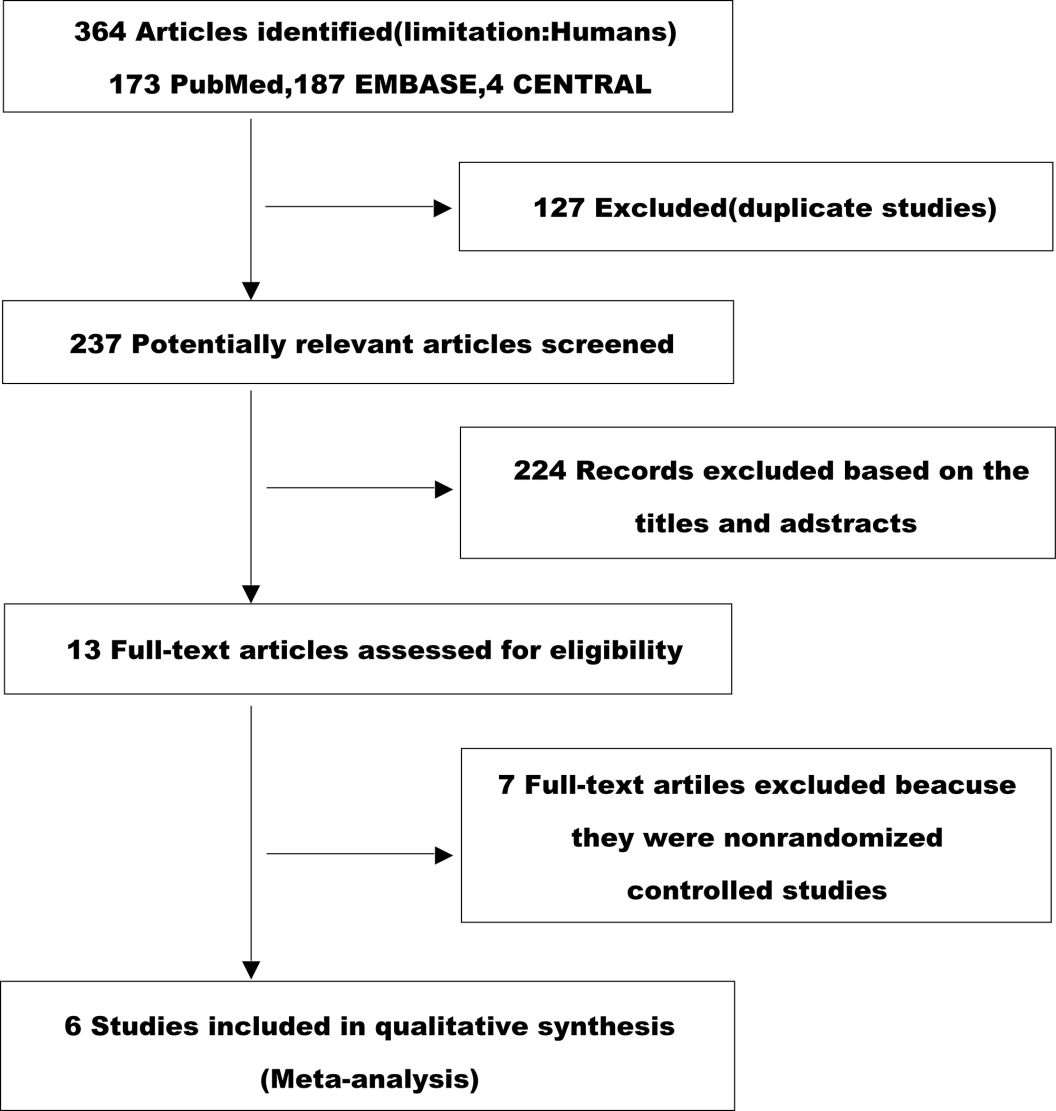
Study Flow Chart. A flow chart showing the process by which studies were selected for inclusion in this systematic review.

After an initial independent search, 364 potentially relevant publications were identified, and 127 duplicates were excluded. The remaining titles and abstracts were reviewed, and 224 publications were excluded because either they were not relevant or they reported the results of non-controlled trials. Of the remaining 13 full-text articles, 7 were excluded because they described nonrandomized controlled studies [13, 20–25]. In the end, 6 articles were included in the present analysis [8, 16, 26–29]. The selection process is shown in Fig 1.

### Study characteristics and quality assessment

Six RCTs were eligible for inclusion. However, while a pleural abrasion group was examined in each of these RCTs, the control groups underwent different interventions, and different outcomes were investigated, as well; therefore, it was not possible to perform a meta-analysis of the data. The benefits of pleural abrasion alone or together with minocycline were evaluated compared with apical pleurectomy in two trials [16, 27]. Another two trials compared the effects of either minocycline pleurodesis or pleural abrasion plus minocycline with that of pleural abrasion alone after VATS [26, 28]. The fifth trial investigated mechanical abrasion with staple line coverage after thoracoscopic bullectomy [29]. The sixth study examined thoracoscopic wedge resection with or without mechanical pleurodesis [8]. Our results are summarized in Table 1.

**Table 1:** Characteristics and quality scores of RCTs included in this systematic review.

Author/year	Location	No. (Abrasion/Control)	Study design	Quality score	PA group	Control group	Length of follow-up (months)
Chen/2006	Taiwan	202 (103/99)	RCT	8	PA after VATS	MP+PA after VATS	29 (range, 12–47)
Rena/2008	Italy	220 (112/108)	RCT	6	PA after thoracoscopic WR	AP after thoracoscopic WR	46 (range, 24–66)
Alayouty/2011	Yemen	82 (40/42)	RCT	6	PA after VATS	MP after VATS	range 28–39
Chen/2012	Taiwan	160 (80/80)	RCT, B, C	9	PA+MP after VATS	AP after VATS	mean 26.1 (range, 12–51)
Lee/2014	Korean	1,414 (657/757)	RCT, C	8	PA after VATS	Staple line covered with absorbable cellulose mesh and fibrin glue after VATS	19.5 (range, 0.3–66.7)
Min/2014	China	289 (145/144)	PG, RCT, C	8	Thoracoscopic WR and PA	Thoracoscopic WR only	18 (range, 6–24)

PSP = primary spontaneous pneumothorax; VATS = video-assisted thoracoscopic surgery; PA = pleural abrasion; AP = apical pleurectomy; MP = minocycline pleurodesis; WR = wedge resection; PG = parallel group; RCT = randomized controlled trial; C = concealed random allocation; B = blinding.

The mean quality score of the studies was 7.5, with a range of 6 to 9 (Table 1). Although none of these trials performed an intention-to-treat (ITT) analysis and the nature of the interventions used for PSP meant that blinding was difficult, only one trial conducted a blinded follow-up of pneumothorax recurrence; in this study, follow-up visits were conducted by a registered nurse who was blinded to the group allocations. The evidence in these studies was of high quality, and they were without overt methodological flaws.

### Pleural abrasion vs. apical pleurectomy

Rena et al. [16] found that pneumothorax recurred in five cases (4.6%) after apical pleurectomy and in seven cases (6.2%) after mechanical pleural abrasion (p = 0.821), based on the results of an RCT that investigated 220 VATS procedures for PSP. In that study, 112 patients underwent mechanical pleural abrasion, and 108 patients underwent apical pleurectomy. The two groups exhibited a similar persistent postoperative air leak rate (5.3% in the pleural abrasion group and 5.5% in the apical pleurectomy group), but post-operative acute bleeding (>200 mL/h for 3h consecutively after the operation) occurred significantly more frequently after apical pleurectomy (8 cases, 7.4%) than after pleural abrasion (1 case, 0.9%; p = 0.036); and the mean operating time was also higher for apical pleurectomy than for pleural abrasion (55±18 versus 38±16 min, respectively; p = 0.0001). Moreover, apical pleurectomy patients experienced significantly more residual chest pain and discomfort compared with pleural abrasion patients (p = 0.001) (Table 2).

**Table 2:** Outcome data of studies included in the systematic review of pleural abrasion for PSP prevention (Abrasion versus Control).

Author/year	Pneumothorax recurrence	Operation bleeding (ml)	Postoperative air leakage	Operating time (minutes)	Residual chest pain	Postoperative pleural drainage	Comments
Chen/2006	8 of 99 vs. 2 of 103 (p = 0.055)	NA	> 5 days: 6 of 99 vs. 2 of 103 (p = 0.100)	76.9±27.3 vs. 81.3±32.6 (p = 0.299)	17 0f 99 vs.16 of 103 (p = 0.753)	Postoperative chest tube duration(days): 3.0± 2.6 vs. 3.0 ±1.2 (p = 0.801)	MP plus PA did not decrease pneumothorax recurrence but tend to decrease the occurrence rate of prolonged air leaks.
Rena/2008	7 of 112 vs. 5 of 108 (p = 0.821)	Post-operative acute bleeding (>200 mL.h-1 for 3 h):1 of 112 vs.8 of 108 (p = 0.036)	> 5 days: 6 of 112 vs. 6 of 108 (p = 0.978)	38±16 vs. 55±18 (p = 0.0001)	VAS of residual chest pain in 180 days: 0.31±0.64 vs. 0.58±0.61 (p = 0.001)	Postoperative chest tube duration (days): 2.53±1.55 vs. 2.92±1.71 (p = 0.065)	No difference in the rate of recurrence was observed. Operating times, postoperative acute bleeding and residual chest pain were greater with AP than with PA.
Alayouty/2011	2 of 40 vs. 0 of 42 (p = 0.000)	NA	Days, mean±SD: 1±5.5 vs. 2±1.1 (p = 0.100)	85±5 vs. 87±3 (p = 0.193)	Comparable but data not available	Comparable but data not available	PSP recurred in two patients after PA and none recurred after MP, but there was no statistical difference.
Chen/2012	3 of 80 vs.3 of 80 (p = 1.000)	13.2±47.8 vs. 29.4±42.2 (p = 0.025)	> 5 days: 2 of 80 vs. 2 of 80 (p = 1.000)	55.8 ± 16.6 vs. 81.4 ± 24.2 (p<0.001)	4 of 80 vs. 6 of 80 (p = 0.514)	Sum of drainage (ml): 195.8± 118.5 vs. 287.4± 309.0 (p = 0.040)	No difference in the rate of recurrence was observed. Patients who underwent AP had a longer operative duration, more bleeding during the operation and more postoperative chest drainage than did patients who underwent PA+MP.
Lee/2014	93 of 657 vs. 99 of 757 (p = 0.555)	NA	> 5 days: 28 of 657 vs. 30 of 757 (p = 0.778)	49.0±19.95 vs. 47.6 ±19.03 (p = 0.17)	Daily medication for residual pain: 21 of 657 vs. 3 of 757 (p<0.0001)	NA	The coverage group and the PA group had the comparable recurrence rate, but the coverage group showed better recovery from pain.
Min/2014	8 of 145 vs. 9 of 144 (p = 0.791)	31 ± 24 vs. 24 ±19 (p = 0.005)	Days, mean± SD: 1.1 ± 0.2 vs. 1.1 ± 0.3 (p = 0.196)	83 ± 40 vs. 83 ± 42 (p = 0.934)	NA	Sum of drainage (ml): 448 ± 327 vs. 371± 279 (p<0.001)	Less intraoperative bleeding and postoperative pleural drainage were observed in the WR-only group, and the postoperative recurrence rate did not differ between the two groups.

PA = pleural abrasion; AP = apical pleurectomy; MP = minocycline pleurodesis; WR = wedge resection; VAS = visual analogue scale (0–10); NA = not available

Chen et al. [27] conducted a randomized, double-blind trial to assess the effects of pleural abrasion with minocycline vs. apical pleurectomy after thoracoscopic stapled bullectomy in 160 patients with a high PSP recurrence risk. They found that the patients in the apical pleurectomy group had longer operative durations (mean, 81.4 minutes vs. 55.8 minutes; p<0.001), more bleeding during surgery (mean, 29.4 mL vs. 13.2 mL; p = 0.025), and greater postoperative chest-drainage volumes (mean, 287.4 mL vs. 195.8 mL; p = 0.040). The patients in the group that underwent abrasion with minocycline had more intense chest pain and required more frequent meperidine injections. Hemothorax occurred in 3 apical pleurectomy patients (3.8%). The short-term results showed that the 2 groups had comparable postoperative chest drainage durations, postoperative hospital lengths of stay, and complication rates. After a mean follow-up period of 26.1 months, ipsilateral pneumothoraces occurred in 3 patients (3.8%) in the pleurectomy group and in 3 patients (3.8%) in the abrasion-with-minocycline group (Table 2). Long-term, residual chest pain and pulmonary function were comparable in both groups.

### Pleural abrasion vs. minocycline pleurodesis

Alayouty et al. [28] designed a study to compare the effectiveness of brushing the pleura vs. instillation of minocycline for the management of PSP and to assess the sensitivity of echography in defining areas of defects. Blebectomy and pleurodesis were performed thoracoscopically on 84 patients (42 patients with abrasion versus 42 patients with minocycline pleurodesis). Echography was performed two weeks after discharge and then repeated two weeks later. Follow-up ranged between 28 and 39 months. Two patients were excluded from the abrasion group due to incomplete follow-up. There was no statistical difference in the pneumothorax recurrence between mechanical pleural abrasion and chemical pleurodesis with minocycline on either an intention-to-treat basis (4 of 42 versus 0 of 42, p = 0.12; Fisher exact test) or after exclusions (2 of 40 versus 0 of 42, p = 0.24; Fisher exact test) (assuming the worst-case scenario of the two mechanical pleurodesis patients lost to follow-up having a recurrence). Likewise, the finding that echocardiography revealed areas of pleural mobility in 5 of 42 mechanical cases versus 3 of 42 chemical cases was not significant (p = 0.71, Fisher exact test). The two groups had comparable chest drainage durations, postoperative hospital lengths of stay and complication rates.

Chen et al. [26] undertook an RCT to compare pleural abrasion alone with pleural abrasion plus minocycline pleurodesis after VATS for PSP. Patients in the abrasion/minocycline group had more intense chest pain and required a higher cumulative dose of meperidine. The short-term results showed that the two groups had comparable chest drainage durations, postoperative hospital lengths of stay and complication rates. Patients in the minocycline group demonstrated a decreased rate of occurrence of prolonged air leaks (1.9% vs. 6.1%, p = 0.100). After a mean follow-up period of 29 months (range, 12–47 months), the pneumothorax recurrence rates of pleural abrasion alone versus pleural abrasion plus minocycline pleurodesis did not exhibit differences (2 of 103 versus 8 of 99, p = 0.055; Fisher exact test). But the authors used Kaplan-Meier estimates of freedom from pneumothorax recurrence and compared the curves using a log rank test (as depicted in their figure 2), then, this approach gave them a p-value of 0.044. However, this is not the same as answering the categorical question of recurrence versus no recurrence. Maybe 6 of the 8 abrasion-only recurrences fell within the first 6 months of follow-up, causing the early separation of the curves. Postoperative long-term residual chest pain and pulmonary function were comparable in both groups.

### Mechanical abrasion vs. staple line coverage

A prospective randomized multicenter study conducted by Lee et al. [29] aimed to determine the efficacy of either an additional staple line coverage procedure or additional mechanical pleurodesis after thoracoscopic bullectomy in preventing the postoperative recurrence of PSP. Following bullectomy performed with staplers, 1,414 patients were randomly assigned to either the coverage group (n = 757), in which the staple line was covered with an absorbable cellulose mesh and fibrin glue, or the mechanical abrasion group (n = 657). The coverage group and the mechanical abrasion group showed the comparable recurrence rate and other surgical outcomes. After a median follow-up period of 19.5 months, the postoperative overall recurrence rates were 13.8% (99/757) in the coverage group and 14.2% (93/657) in the abrasion group. The overall recurrence rates for PSPs that required intervention were 8.1% (61/757) in the coverage group and 10.2% (67/657) in the mechanical abrasion group. But patients in the mechanical abrasion group had significantly more residual pain (3.2% vs. 0.4%, p<0.0001) (Table 2).

### Thoracoscopic wedge resection and mechanical pleurodesis vs. wedge resection alone

Min et al. [8] conducted a prospective, parallel-group RCT in 289 patients at 2 hospitals in China. The patients were randomly assigned (1:1) to receive thoracoscopic wedge resection only (WR group) or thoracoscopic wedge resection together with mechanical pleurodesis (WR+MP group). Significantly less intraoperative bleeding (p = 0.005) and postoperative pleural drainage (p<0.001) occurred in the WR group. The postoperative recurrence rate did not significantly differ between the groups (log-rank test, p = 0.791; Breslow test, p = 0.722).

## Discussion

Mechanical pleurodesis has been widely performed to prevent the recurrence of PSP as an adjunct to wedge resection of bullous lesions. Mechanical pleurodesis results in adhesion between the visceral and parietal pleura by mechanical irritation or partial removal of the parietal pleura. Either apical pleurectomy or parietal pleural abrasion can be performed for mechanical pleurodesis [8]. Videothoracoscopy is a rapidly developing technique that allows many surgical procedures to be performed without thoracotomy. VATS enables us to inspect the entire lung, identify bullae, and resect the bullous tissue. Pleural abrasion can be performed via VATS due to its simplicity, its short operative time, and the ease with which it can be performed [13]. However, the current role of pleural abrasion in the treatment of PSP remains unclear; the six RCTs included in our systematic review are of high methodological quality, but the heterogeneity of the interventions and outcomes in the control groups does not allow a meta-analysis of the data to be conducted.

Many surgeons perform additional procedures, such as parietal pleural abrasion or pleurectomy, after wedge resection to minimize postoperative PSP recurrences [29]. Apical pleurectomy is generally thought to effectively prevent the recurrence of PSP [30]. Several studies have also shown that apical pleurectomy is a reliable pleurodesis procedure that is associated with a lower rate of recurrence than is pleural abrasion [22, 23, 31]. Two retrospective studies have reported that the PSP recurrence rate with pleural abrasion was higher than that with apical parietal pleurectomy [13, 23], but the different numbers of patients and follow-up durations make the results of these studies unconvincing. To generate robust results in the present systematic review, we included only RCTs that clearly stated that patients were enrolled to compare mechanical pleural abrasion with apical pleurectomy for the treatment of PSP. In one RCT [16], the patients with PSP treated with mechanical pleural abrasion or apical pleurectomy exhibited similar persistent postoperative air-leak rates and recurrence rates at 1 year, whereas hemothorax was significantly more frequent after apical pleurectomy than after pleural abrasion; and the mean operating time was also higher for apical pleurectomy than for pleural abrasion. Moreover, apical pleurectomy patients experienced significantly greater residual chest pain and discomfort than did pleural abrasion patients. In another RCT [27], patients treated with either pleural abrasion with minocycline or apical pleurectomy showed the same pneumothorax recurrence rate, although the intensity of chest pain was greater in the patients treated with abrasion with minocycline. However, patients in the pleurectomy group had longer operative durations, more bleeding during surgery and greater postoperative chest drainage volumes. These findings suggest that pleural abrasion is as effective as apical pleurectomy in preventing pneumothorax recurrence but has fewer complications and that the chest pain may be due to minocycline. The main finding of our systematic review is inconsistent with the results of a previous meta-analysis, which showed that pleural abrasion tended to be associated with a high relative risk of pneumothorax recurrence compared with pleurectomy, although the overall effect was not statistically significant [32]. However, the four RCTs in the previous meta-analysis did not randomly compare pleurectomy with pleural abrasion, and all of the trials were published before October 2006 [33–36].

In clinical practice, a variety of sclerosants has been widely applied to prevent pneumothorax recurrence, including tetracycline and its derivatives (doxycycline or minocycline), talc, bleomycin, autologous blood patches, iodopovidone, picibanil, silver nitrate, and quinacrine [37–42]. The present systematic review of RCTs examined the effects of minocycline pleurodesis with or without pleural abrasion for preventing pneumothorax recurrence. Chen et al. [26] compare pleural abrasion alone with pleural abrasion plus minocycline pleurodesis after VATS for PSP, they found that the pneumothorax recurrence rates of pleural abrasion alone versus pleural abrasion plus minocycline pleurodesis did not show any differences, moreover, abrasion/minocycline group had more intense chest pain and required a higher painkiller. Alayouty et al. [28] also found that there was no statistical difference on the pneumothorax recurrence between mechanical pleural abrasion and chemical pleurodesis with minocycline. It appears that minocycline pleurodesis, with or without pleural abrasion, is not safer and more effective than pleural abrasion alone.

Some procedures have been performed by covering the surgical pulmonary margins with absorbable mesh and fibrin glue to reinforce the visceral pleura with a goal of decreasing the postoperative recurrence of pneumothorax [43, 44]. Lee et al. [29] found that the postoperative overall recurrence rate of visceral pleural coverage with absorbable cellulose mesh and fibrin glue after thoracoscopic bullectomy was similar to that of mechanical pleural abrasion. However, the coverage group exhibited significantly lower residual pain (p<0.0001).

The aims of surgical treatment for spontaneous pneumothorax are the closure of the site of the air leak, facilitation of the complete expansion of the lungs, and the prevention of future recurrences [45–47]. Some have questioned the efficacy of wedge resection alone by VATS in the treatment of spontaneous pneumothorax because of the high recurrence rate following this procedure; hence, various pleural procedures have been performed to reduce this recurrence rate. However, a retrospective study [11] of 263 patients found that thoracoscopic wedge resection alone successfully controlled PSP and that additional pleural abrasion did not decrease the recurrence of pneumothorax after wedge resection of bullae for PSP. Moreover, younger age was associated with a higher risk of recurrence (p<0.0001). The main finding of our review appears to be consistent with these findings. In a RCT, Min et al. [8] showed that thoracoscopic mechanical pleurodesis after wedge resection did not significantly decrease pneumothorax recurrence compared with simple wedge resection, but intraoperative bleeding and postoperative pleural drainage rates were increased with mechanical pleurodesis after thoracoscopic wedge resection. This suggests that the use of pleural abrasion after thoracoscopic wedge resection for the treatment of PSP may not be recommended.

Several limitations of this systematic review should be noted. First, this study examined six RCTs that compared the effects of pleural abrasion with those of different interventions; thus, the pneumothorax recurrence rate in the control group was different in each study, and these differences made it difficult to compare the advantages and disadvantages of the procedures used in each trial. Second, the follow-up time varied among the included studies, and these differences could potentially have impacted our results. Finally, it is possible that the exclusion of some missing and unpublished data led to a bias in the effect size; the exclusion of non-English-language studies may also have led to publication bias.

In conclusion, a systematic review of RCTs suggests that thoracoscopic pleural abrasion with or without minocycline pleurodesis is safer than apical pleurectomy and is associated with the same pneumothorax recurrence rate. Currently, there is no evidence that minocycline pleurodesis with or without pleural abrasion is any more effective than pleural abrasion alone. Moreover, additional mechanical abrasion is not safer than additional staple line coverage with absorbable cellulose mesh and fibrin glue after thoracoscopic bullectomy because of the increase in postoperative pain, and pleural abrasion after thoracoscopic wedge resection should not be recommended for routine application due to the greater risk of adverse effects than wedge resection alone. However, further large-scale, well-designed RCTs are needed to confirm the best procedure.

## Supporting Information

S1 Checklist A PRISMA checklist for this systematic review. (DOC)
